# Emulsion-Based Multicompartment Vaginal Drug Carriers: From Nanoemulsions to Nanoemulgels

**DOI:** 10.3390/ijms22126455

**Published:** 2021-06-16

**Authors:** Michał Smoleński, Bożena Karolewicz, Anna M. Gołkowska, Karol P. Nartowski, Katarzyna Małolepsza-Jarmołowska

**Affiliations:** Department of Drug Form Technology, Wroclaw Medical University, Borowska 211 A, 50-556 Wroclaw, Poland; michal.smolenski@student.umed.wroc.pl (M.S.); bozena.karolewicz@umed.wroc.pl (B.K.); anna.golkowska@student.umed.wroc.pl (A.M.G.); karol.nartowski@umed.wroc.pl (K.P.N.)

**Keywords:** vaginal formulations, vaginal drug delivery, emulsion-based dosage forms, vaginal administration, drug carriers for gynecology

## Abstract

In order to overcome the limitations associated with vaginal administration of drugs, e.g., the short contact time of the drug form with the mucosa or continuous carrier wash-out, the development of new carriers for gynecological use is necessary. Furthermore, high individual anatomical and physiological variability resulting in unsatisfactory therapeutic efficacy of lipophilic active substances requires application of multicompartment drug delivery systems. This manuscript provides an up-to-date comprehensive review of the literature on emulsion-based vaginal dosage forms (EVDF) including macroemulsions, microemulsions, nanoemulsions, multiple emulsions and self-emulsifying drug delivery systems. The first part of the paper discusses (i) the influence of anatomical-physiological conditions on therapeutic efficacy of drug forms after local and systemic administration, (ii) characterization of EVDF components and the manufacturing techniques of these dosage forms and (iii) methods used to evaluate the physicochemical and pharmaceutical properties of emulsion-based vaginal dosage forms. The second part of the paper presents (iv) the results of biological and in vivo studies as well as (v) clinical evaluation of EVDF safety and therapeutic efficacy across different indications.

## 1. Introduction

The vaginal route of drug administration has been commonly used for many years in contraceptive delivery methods and the treatment of local vaginal infections in the form of vaginal solutions, ointments, pessaries, rings, suppositories and tablets [1]. However, in order to overcome limitations related to intravaginal drug administration, i.e., short contact time with mucosa, constant washing-out by vaginal discharge, low volume of vaginal discharge and high individual anatomical and physiological variability, there is a need for novel gynecological formulations development, such as hydrogels, films, micro- and nanoemulsions or nanoparticles-based drug delivery systems [2,3,4,5,6,7,8,9,10]. The vaginal route as a potential way for systemic drug delivery has also been considered [11]. Nonetheless, there are several limitations affecting drug bioavailability after intravaginal application related to physiological factors of female genitals and physicochemical properties of the drug itself [11]. The drug absorption process from the vaginal lumen has two main steps—drug dissolution in the vaginal discharges and the mucosa drug penetration stage [12]. The ideal vaginal product should be water soluble, able to penetrate biological membranes and be resistant to washing out. As a result, most technological studies are focused on the development of easy to administer, highly mucoadhesive formulations [3,10,13]. Emulsions, as multi-compartment preparations, in contrast to conventional hydrophilic formulations, allow the simultaneous vaginal application of hydrophilic and lipophilic substances, reducing the risk of pharmaceutical incompatibilities. Furthermore, emulsion-based vaginal dosage forms (EVDF) in comparison to hydrophilic carriers exhibit favorable formulation properties for vaginal administration, i.e., increased retention time at the application site and controlled penetration of active substances through the vaginal mucosa [2,3,5,6,10].

This review aims at providing the readers with a comprehensive analysis of the composition, manufacturing methods and evaluation methodology of emulsion-based vaginal dosage forms as well as the therapeutic effects achieved after administration of these formulations. For this purpose, Scopus and Web of Science databases were searched, using the intravaginal, vaginal, gynecological, emulsion, microemulsion, nanoemulsion, multiple emulsion, SEDDS (Self-Emulsifying Drug Delivery Systems), *vagin* and *emuls* keywords and limiting the period time from 2000 to 2020. Examples of EVDF have already been briefly discussed in the context of mucoadhesion and vaginal formulations, however, to the best of our knowledge, this is the first review which provides the current status of multicompartment emulsion-based vaginal dosage forms, enabling this group of colloidal formulations to stand out from other available topical formulations [3,4,10,14,15].

## 2. The Anatomical and Physiological Aspects Intravaginal Drug Application

### 2.1. Vaginal Anatomy

**Figure 1:**
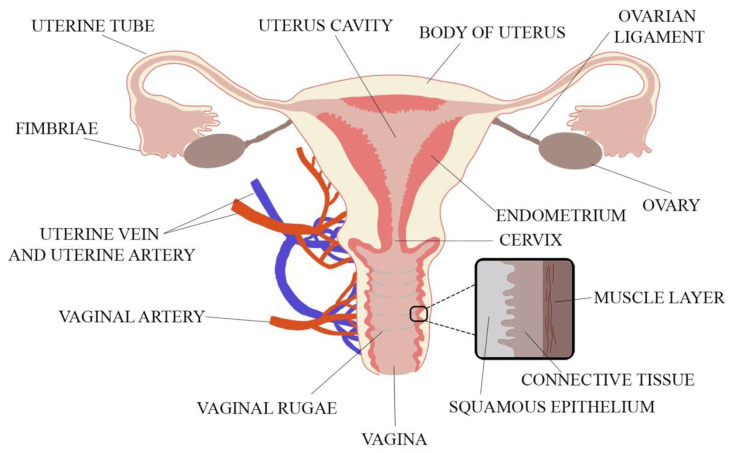
The vaginal anatomy with its wall structure (adapted from [18]).

When designing a novel vaginal drug form, anatomical and physiological aspects must be considered. These include the properties of the vaginal environment (e.g., pH, volume of vaginal discharge or the presence of microorganisms), its total inner surface, the vaginal vascularization and mucus structure. The vagina is a part of the female internal genitalia, located in the pelvis minor and described as an extensible, collapsible, fibromuscular, curved tube connecting the uterus (cervix) with the vulval vestibule [16,17]. The vagina is attached at its upper end to the uterus above the cervix. The spaces between the vagina and the cervix called fornices consist of anterior, lateral and posterior parts [18]. The vagina is 6–12 cm long and 2.1–5.0 cm wide. As the posterior vaginal wall is attached to the uterus higher than the anterior wall, the length of the posterior part is 8–12 cm and the length of the anterior part is 6–9 cm. Vaginal length is age-dependent and shortens by ca. 0.08 cm per 10 years with menopause enhancing this process [18,19]. Vaginal walls are in apposition so that in the cross-section vaginal lumen is centrally flattened and broadens towards the ends, and thus, the cross-section of vagina reminds the shape of letter H [19]. The H-shaped cross-section of the vagina comes from central flattening of the vaginal lumen with broadening towards the ends. The thin vaginal wall consists of three layers. The inner layer is the vaginal mucosa formed of nonkeratinized, squamous epithelium which is connected to the middle layer—lamina propria—composed of collagen and elastic tissue with significant vascular and lymphatic circulation surrounded by the smooth muscle coating. The third layer is the outer fibrous layer—tunica adventitia—which contains large plexus of blood vessels [1,18,19]. The anatomy of the female reproductive system is shown in Figure 1.

### 2.2. Vascularization of the Vagina

The vagina has a vast anastomotic vascular system which is connected with the uterine artery, branch of the internal iliac artery or vesical or rectal venous plexuses. Extensive vascular networks from the middle and upper regions of the vagina are connected to inferior vena cava bypassing hepatic blood circulation which, in consequence, lowers the impact of hepatic metabolism (i.e., first pass effect) on the drug plasma concentration after intravaginal application [12,19]. On the other hand, vast connections of the vaginal vascular system with the uterus are responsible for the so-called first uterine pass effect (FUPE) and accumulation of the drug in the uterus. FUPE is well described for hormones, i.e., measured concentrations of progesterone are higher in endometrial tissues and lower in blood plasma after intravaginal administration in comparison to intramuscular injections [20]. The described phenomena may prove beneficial in the treatment of uterus cancer by reducing the risk of serious side effects related to high anticancer drug plasma concentrations after systemic application. On the other hand, uterus accumulation of narrow therapeutic index drugs (NTI-drugs), e.g., cytostatic agents administered in the adjuvant therapy of cancer, may have a toxic effect and lead to serious side effects [7,21]. The vaginal application could also be considered as a route of systemic drug administration due to rich vascularity of the vagina [11]. Wing et al. [22] studied intravaginal delivery of misoprostol and proved higher efficiency and fewer side effects in the induction of labour and ripening of cervix compared to oral administration.

### 2.3. Vagina Surface Area

The unique physiological environment prevailing inside the vagina and the vaginal surface properties determine intravaginal formulations requirements for spreadability, extensibility and extended contact time of the drug with the mucosa. The total vaginal inner surface area varies across individuals, wherein the volume of vaginal lumen limits the maximum applicable dose. Early measurements and estimations of vaginal surface area displayed large discrepancies (50 and 600 cm^2^) that are now narrowed to ca. 360 or 390 cm^2^ depending on the measuring method [14]. Differences arise from the increase of vaginal mucosa surface in reproductive-aged women by the folds and microridges called the rugae which cover epithelial cell surface especially in the lower third of the vagina. The size of vaginal epithelium changes additionally during the menstrual cycle due to estrogen sensitivity. In summary, the formulation contact surface area with vaginal mucosa depends on individual factors, e.g., age, stage of the menstrual cycle and depth of application into the vagina, due to the presence of folds in different parts of the organ [1,10,18,19].

### 2.4. Vaginal Mucosa and Vaginal Discharge

Another factor influencing the drug absorption process after intravaginal application is the three-layer construction of the vaginal wall and the presence of an internal mucosa layer deprived of secretion glands. A thin layer of vaginal discharge (ca. 1–2 mL) on the surface of vaginal walls is constantly present providing the lubrication for the vagina. However, no exact data on ambient vaginal discharge volume is available. Mean production of vaginal secretions is ca. 1.5–2.0 mL per 8 h, up to 6.0 mL per day. The vaginal discharge volume is directly proportional to the estrogen level and inversely proportional to the progesterone level [23]. Furthermore, sexual stimulation enhances the production of vaginal discharge up to 2.8 mL per hour [24]. The discharge origins in the vaginal venous plexus, where it is secreted by the cervical and Bartholin glands through transduction, and is primarily composed of water, 1–2% of mucin and electrolytes including sodium (Na^+^) and potassium (K^+^) [14,18,19,24]. Mucin is one the most abundant glycoprotein in the vaginal mucus layer and it is responsible for the gel-like mucus mesh properties formed from negatively charged mucin fibers. Average vaginal discharge pH is between 3.8 to 4.5, however other reports expand the normal pH value of vaginal discharge from 3.4 to 6.4, with an average pH at 4.7 [19]. The acidity of the vaginal environment is controlled by the constant secretion of lactic acid by *Lactobacillus* spp. bacteria [25]. Vaginal discharge pH may be affected by age, hormonal stimulation during menstruation, menopause, pregnancy, inflammations, composition of vaginal microbiota, presence of pathogenic microorganism and even ethnical origin and race [24,26,27,28,29,30,31,32]. The presence of vaginal discharge on the mucosal surface, including its quantity and pH, influences the drug dissolution as well as passive and active drug transport through the vaginal membrane [1,33]. Vaginal discharge pH level affects drug dissociation as pH regulates the ionization of active substances and, in consequence, has an impact on permeation of non-ionized drug through the vaginal mucosa [33]. Passive and active drug absorption processes from the vaginal lumen occur not only via transcellular and paracellular routes, but also by the means of vesicular and receptor-mediated transport [1,11,33]. In general, after vaginal application, lipophilic molecules are transported by the transcellular route, while hydrophilic substances are absorbed by paracellular diffusion. Low molecular weight lipophilic drug molecules undergo better absorption than the larger ones or hydrophilic compounds [33]. Constant secretion of the vaginal discharge and its high viscosity form a strong diffusional barrier for drug absorption [11]. In order to achieve high permeability crucial for systemic administration of a drug, low molecular weight lipophilic APIs (active pharmaceutical ingredients) should be encapsulated in water-based formulations due to hydrophilic vaginal environment and small ambient volume of vaginal discharge. Increased quantity of the discharge could enhance poorly water-soluble drugs absorption, on the other hand, the constant fluid secretion could cause the decrease of API bioavailability as a result of formulation wash-out.

### 2.5. The Influence of Vaginal Microbiota

The vaginal microbiota controls the acidity of vaginal secretions through the lactic acid production [25]. The most abundant group of vaginal microbiota are the *Lactobacillaceae* spp. (28.1%), followed by *Bifidobacteriaceae* spp. (10.1%) and *Prevotellaceae* spp. [34]. *L. crispatus* and *L. iners* are the most commonly reported within *Lactobacillus* spp. in women of reproductive age, but *L. jensenii* and *L. gasseri* presence has also been described [35,36]. In most cases one or two of *Lactobacillus* species, rather than many different species, are isolated from the vagina of a particular patient [36]. The vaginal *lactobacilli* apart from acidification have the ability to produce hydrogen peroxide and bacteriocins which eradicate pathogenic microorganisms [37]. Composition of vaginal microbiota undergoes dynamic changes as a consequence of ageing, pH changes, hormonal status, ethnicity, genetic background and exogenous factors, i.e., administered medicines, contraceptive method as well as sexual, behavioral and hygiene practices, diet or stress [38,39]. There are variances in the pregnant and non-pregnant women microbiota composition which can be used for early prediction of pre-term births [40,41,42]. Slight changes in microbiota composition were also observed in patients with deep endometriotic lesions [43].

## 3. Technological Aspects of Vaginal Formulations Development

Based on the anatomical and physiological aspects of the intravaginal drug administration route, the optimal intravaginal formulation should (a) enable the delivery of low molecular weight hydrophilic or lipophilic compounds as they have the highest absorption rates due to their transport mechanism; (b) be partially water-soluble due to the aqueous properties of the vaginal discharge; (c) be easy to administer; (d) have bioadhesive properties in order to prevent washing-out from the vaginal lumen [14,44]. Another influential property of an intravaginal drug formulation is its osmolarity. Based on WHO recommendations for lubricants [45], the intravaginal formulations should have pH of about 4.5, while their osmolarity should be below 1200 mOsm/kg and ideally not exceed 380 mOsm/kg. Machado et al. analyzed the osmolarity of market-available semi-solid formulations, i.e., creams and gels for intravaginal drug delivery with antifungal azoles, antibacterials and estrogens [46]. Authors concluded that hypotonic formulations < 260 mOsmol/kg increase particles penetration through the mucus layers leading to improved and rapid drug absorption. These formulations are also considered safer for vaginal epithelium due to the reduction of time needed to obtain the effective drug concentration and therapeutic effect [46,47]. It has been reported that hyperosmolar vaginal dosage forms with microbicide could cause vaginal irritations, increase toxicity and infection susceptibility to, e.g., HSV-2 [47,48].

Size of drug carrier particles in the formulation also affects the penetration of APIs through the mucus layer [14,49]. Particles within the range of 200–500 nm penetrate cervicovaginal mucus in a more efficient way than particles sized around 100–150 nm. In turn, particles smaller than 100 nm enter pocket-like mucus mesh channels where they are immobilized. Larger particles with sizes ranging 200–500 nm are too big to enter the pocket-like channels, so they penetrate mucus mesh more rapidly [49,50,51]. Frey et al. and Ponchel et al. [52,53] found that particles greater than 1000 nm are not able to enter channels in the gastrointestinal mucus mesh. The studies on vaginal mucus mesh are limited, however structural similarities between different types of mucus have been shown, hence the possible application of these findings in relation to vaginal mucus mesh. Additionally, not only the size of drug vehicles matters but also the chemical composition of the vehicle surface [14]. Lipophilic drugs are immobilized in the mucus mesh due to the electrostatic and hydrophobic interactions between mucins fibers and lipophilic vehicles. The nature of this phenomenon was analyzed by Lai et al. and Wang et al. by anchoring hydrophilic and neutrally charged motifs on a hydrophobic polystyrene surface. In this case addition of hydrophilic polymers forms a “slippery surface” by the reduction of hydrophobic interactions between the hydrophilic PEG-coated particles and the hydrophobic mucin fibers, resulting in rapid penetration of the particles through the cervicovaginal mucus [49,54,55]. Ensign et al. also highlighted hydrophobic interactions as a barrier to nanocarriers [51].

The bioadhesion of a vaginal formulation to the vaginal mucus can be enhanced by the addition of positively charged polymers, e.g., N-trimethyl chitosan. This process takes advantage of electrostatic attraction between the negatively charged mucin and the positively charged polymer particles. Enhanced binding to the mucins results in an increased resistance to formulation wash-out after application [56,57]. The summary of optimal API and formulation properties is presented in Table 1.

### 3.1. Emulsions–Based Vaginal Dosage Forms (EVDF)

In 1972 Everett defined O/W emulsion as disperse systems, where *the continuous phase is an aqueous solution* (W) in contrary to W/O, where a continuous phase is an oil or other organic liquid (O) [58]. Following the IUPAC Gold Book, an emulsion is defined as *a fluid colloidal system in which liquid droplets and/or liquid crystals are dispersed in a liquid* [59].

The addition of surfactants and cosurfactants (S_mix_) is required to obtain such colloidal systems. Emulsions are classified according to their droplet size (macro- vs. microemulsions) and composition of both continuous and inner phase (e.g., O/W or W/O). However, thermodynamic and kinetic stability needs to be considered while distinguishing between micro- and nano-emulsions [60,61,62,63,64,65,66,67,68,69,70,71]. Pre-emulsions, on the other hand, are defined as a self-emulsifying drug delivery system (SEDDS), forming emulsion in situ after dilution with aqueous media [72]. The terms self-emulsifying drug delivery system (SEDDS), self-microemulsifying drug delivery systems (SMEDDS) or self-nanoemulsifying drug delivery systems (SNEDDS) might be questionable from a physical point of view as spontaneous formation of an emulsion is a characteristic of a microemulsion and low-energy emulsification might be misinterpreted as a self-emulsification process [67].

In this review we use ‘SEDDS’, ‘SMEDDS’ and ‘SNEDDS’ following the definition proposed by Pouton as pre-formulations of an isotropic mixture of oils and surfactants which form emulsions after contact with body fluids [72]. The classification and characteristics of emulsion-based dosage forms are presented in Table 2.

**Figure 2:**
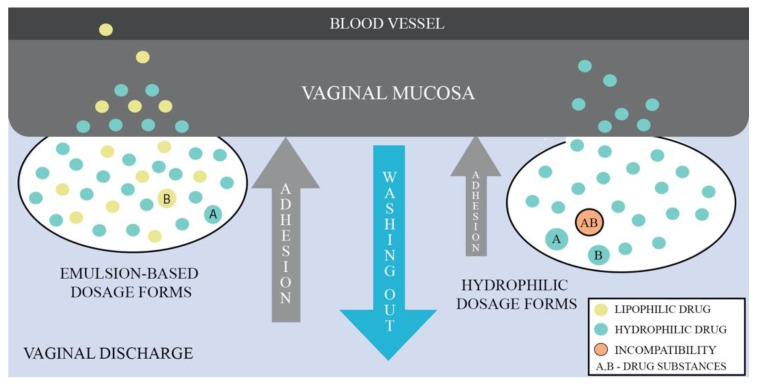
Comparison of vaginal emulsion-based and hydrophilic drug dosage forms.

Given that emulsions consist of two phases, aqueous and oil, they have the ability to deliver lipophilic and hydrophilic APIs simultaneously. Depending on the type of emulsion, it is possible to obtain droplets size in the range of nano- to micro-meters enabling to control mucus barrier penetration. The drug membrane penetration can also be enhanced by the use of medium- and long-chained mono/triglycerides as the oil phase or an addition of nonionic surfactants and cosurfactants. Additionally, incorporation of API into the lipid phase has drug-protective properties against enzymatic components of mucus and fluids [73]. Figure 2 shows a comparison of the behavior between emulsion-based dosage forms and hydrophilic forms when administered vaginally, demonstrating the advantages of the emulsion-based dosage forms.

### 3.2. The Technological Aspects of Emulsion-Based Vaginal Dosage Forms

#### 3.2.1. The Vaginal Drug Dosage Form Compositions

Emulsion-based vaginal dosage forms are composed of dispersed (lipids, oils) and continuous (aqueous solutions) phases, surfactants, cosurfactants and other excipients required to control pharmaceutical properties of the formulation (see Table 3).

##### Oil Phase

Several oils have been proposed as dispersed phase in EVDF formulations including vegetable oils (i.e., copaiba oil or soybean oil), mineral oils (i.e., paraffin oil) [74], and essential oils, sterols [75], phospholipids [76,77], fatty acids, fatty acid esters, poly alcoholic fatty acid esters, alkene derivatives and organosilicon compounds (Table 3) [78,79]. Organosilicon compounds, i.e., cyclomethicone tetramer and pentamer, were used in manufacturing of water-in-silicone (W/S) macroemulsions enabling controlled release of the API from the formulation and enhanced washing-out by vaginal secretion resistance [80,81].

For the preparation of vaginal microemulsions and SEDDS systems, fatty acid derivatives such as Captex^®^ 300 (ABITEC, Columbus, USA), Capryol^®^ 90 (Gattefossé, Saint-Priest, France), glycerol monolaurate and oleic acid have been used as the oil phase components [76,77,82,83,84,85,86,87,88]. These excipients exhibited high solubility of lipophilic compounds, e.g., clotrimazole or tetrahydrocurcumin and were also selected due to their additional properties, such as strong microemulsifying properties (Capryol^®^ 90, Gattefossé, Saint-Priest, France), anti-HIV activity (glycerol monolaurate) and low toxicity (Captex^®^ 300, ABITEC, Columbus, USA) [76,77,82,83,84,85,86,87,88]. Cetyl palmitate and other diesters and triesters of fatty acids, e.g., Labrasol^®^ (Gattefossé, Saint-Priest, France), triglycerides of medium-length fatty acids have also been proposed in the preparation of vaginal emulsions [76,79,82,83,84,85,86,89,90,91,92]. The medium- and long-chain mono/triglycerides are frequently utilized as the oil phase due to their advantageous safety profile and enhanced absorption of the active substance upon application to mucous membranes [73]. Furthermore, the essential oils, e.g., tea tree oil, eucalyptus oil, geranium oil, mint essential oil from *M. spicata* var. *virdis* and lemongrass oil, can act as multifunctional excipients due to their antimicrobial properties [93,94,95,96].

##### Surfactants

Surfactants are amphiphilic chemical compounds enabling the decrease of surface and interfacial tension, i.e., the tension that forms at the interface between immiscible phases of colloidal systems [58]. Surfactants adsorb at the hydrophilic and hydrophobic phase interface, depending on their chemical structure, allowing to obtain different types of O/W or W/O emulsions. The size of the droplets, degree of polydispersity (defined as polydispersity index, PDI) as well as the kinetic and thermodynamic stability can be controlled via carefully selected mixture of surfactants and cosurfactants. The surfactants are usually classified according to their experimentally determined hydrophilic–lipophilic balance (HLB, in the range of 0–20), which is structure-based. The higher the HLB value, the more hydrophilic the surfactant. Surfactants with HLB value 0–7 form W/O emulsions, whereas wetting agents with HLB value >7 form O/W emulsions [107]. Apart from the surfactant’s chemical nature, the properties and type of the obtained emulsion are influenced by the surfactant concentration in the formulation, the so-called surfactant-to-oil ratio (SOR). In nanoemulsions containing oils of natural origin, SOR value is usually <2, whereas in the case of microemulsions, which require a steeper reduction in surface tension, the SOR is often higher (usually >2), as a result of lower ability of these systems to incorporate the dispersed phase [108,109]. Rao et al. in their study homogenized a formulation composed of lemon oil, Tween 80 and water using low, medium and high SOR values and obtained different types of emulsion systems: macro-, nano- and microemulsions, respectively [109]. The higher surfactant concentration in formulations can extend the physical stability of the emulsion, but often at the cost of reducing the hydrophilic or lipophilic phase content. In water-in-silicone oil (W/S) emulsions (see Table 4) characterized by high interfacial tension between the silicone and aqueous phases an increase in the concentration of the surfactant in the system results in a size decrease of dispersed phase droplets and an increase in their homogeneity. It occurs until a critical value of dispersed droplets size is reached for a given composition, above which further increase of the surfactant content in the formulation does not change size and homogeneity of a W/S emulsion [110].

The increase of surfactant content at the cost of the hydrophilic phase may be compensated after vaginal application of small volumes of the formulation that is diluted with the secretions present in the vaginal lumen (ca. 1–2 mL) [23]. When low SOR emulsions are obtained it is possible to use a higher ratio of the oil phase, thus increasing the hydrophobic API content in the formulation and enabling for a dose decrease.

When establishing the surfactant composition for EVDF additional aspects such as the API’s solubility in the chosen excipient composition and their safety profile should be considered. In order to obtain the highest possible concentration of poorly soluble active substances in the formulation the solubility studies of an API are conducted using mixtures of oil and surfactants. This may consequently be a determining factor for the choice of surfactants and cosurfactants, especially if these systems have a high SOR value [79,82,84,85,90,92,97,98,99]. As surfactants are used as spermicides with vaginal mucosa irritating properties [100], analysis of their safety profile after the vaginal application is another important criterion in the surfactant selection process. Some of the well-known non-ionic and ionic surfactants such as benzalkonium bromide, nonoxynol-9, sodium dodecyl sulphate and Triton X-100 have potential mucosal irritating activity and are rarely used in gynecological formulations [111,112,113]. Additionally, some surfactants, such as Capmul MCM, have an inhibitory effect on the growth of endogenous vaginal bacterial flora creating a risk of potential infections [114]. On the other hand, advantageous properties of surfactants such as the API permeation enhancement through the vaginal mucosa have been well-documented in the case of Polysorbate 80 and Labrasol and can be employed to improve the therapeutic efficacy of the administered drugs [73]. The list of surfactants used as components of EVDF is summarized in Table 3.

Multiple emulsions are a specific case of emulsion systems whose manufacturing requires both lipophilic and hydrophilic surfactants (see Table 5). A non-ionic silicone based Abil EM 90 W/O emulsifier is the most commonly used lipophilic surfactant in the formation of internal emulsion, while a hydrophilic surfactant, e.g., poloxamer 407 provides w/o/w multiple emulsion stability by encapsulation of w/o emulsion in external aqueous phase [89,101,102,103,104]. In turn, obtaining SEDDS formulations is a difficult process due to the limited number of oil-surfactant combinations capable of forming these systems (see Table 6). This process requires numerous preliminary tests to select the type and mutual proportion of these components [115].

##### Cosurfactants

Carefully selected cosurfactants can increase the stability of the obtained emulsion, reduce the size of the emulsion droplets and the required concentration of surfactant in the formulation. Cosurfactants increase the ability of the oil-surfactant system to emulsify the aqueous phase and further reduce the tension present at the hydrophilic-lipophilic interface. An example of the beneficial effect of ethylene glycol used as a cosurfactant in combination with the Cremophor EL in microemulsions and nanoemulsions with ethyl oleate as an oil phase is the achievement of a twofold reduction of the inner phase droplet size and a significant reduction of PDI compared to formulations obtained without the addition of the cosurfactant [116,117]. The commonly used cosurfactants in the EVDF include (Table 3): short-chain mono- and polyhydric alcohols [79,83,85,86,90,92,98], soybean phosphatidylcholine [75], fatty acids and fatty acid esters [91,105], polyethylene glycols and their derivatives [74,94,95,99,105] and polysorbates [78,93,97].

##### Other Excipients

In emulsion-based vaginal dosage forms several other excipients such as gelling agents, electrolytes, pH regulators, humectants and preservatives (also acting as antiseptics) are frequently employed to modify pharmaceutical properties of the formulation. The addition of gelling polymers, i.e., carbomer, chitosan, hydroxypropyl methyl cellulose (HPMC), methyl cellulose (MC), sodium carboxymethyl cellulose (NaCMC), Pluronic F127, xanthan gum to the aqueous phase increase formulation viscosity enabling for improved stability, emulsion adhesiveness and contact time at the application site [76,77,82,84,85,86,89,90,92,93,94,95,98,118,119,120,121,122]. The increased viscosity of the formulation facilitates homogeneous dispersion of the droplets in the continuous phase, limits the mobility of the oil droplets and, as a consequence, prevents flocculation and creaming processes [118,119,120,121,122]. The addition of a gelling polymer results in the transformation of an emulsion into an emulgel, increasing the system’s resistance to pH level changes and oxidation [123]. The microemulgels have been the most commonly used forms among microemulsion-based vaginal dosage forms [76,77,82,84,85,86]. The addition of polymers to microemulsions reduces the mobility of the continuous aqueous phase, decelerating potential destabilization processes resulting from temperature changes and enhanced vaginal secretions after administration [67,68,124]. In multiple emulsions and SEDDS-type systems (Table 5 and Table 6) the addition of gelling polymers is limited [89,106] as minor addition of an electrolyte into the internal aqueous phase of a multiple emulsion often increases the viscosity of the formulation [125]. The electrolytes influencing the stability and rheological properties of vaginally administered multiple emulsions include magnesium sulphate or sodium chloride [89,102,104].

Among other excipients used in vaginal emulsion systems the addition of lactic acid or phosphate buffers for pH regulation were reported [75,92,101], while propylene glycol and PEG 200 have been applied as humectants [77]. Benzyl alcohol, sodium benzoate, chlorocresol, methylparaben are used as preservatives [82,84,95]. The absence of preservatives in the formulation reduces the risk of vaginal mucosal irritation. On the other hand, many emulsion-based vaginal dosage forms are in the preliminary research phase, and preservatives use may only be purposeful after their full clinical evaluation of preformulation selected for further development.

#### 3.2.2. EVDF Preparation Methods

**Figure 3:**
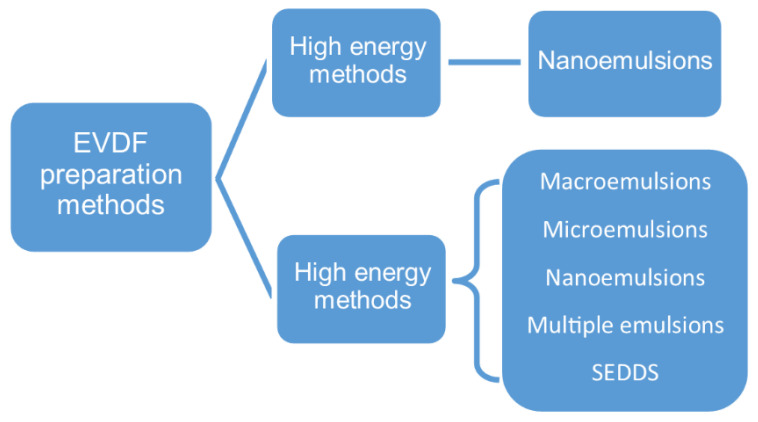
EVDF preparation methods.

An emulsion can be formed in a free energy associated process without the application of mechanical forces or energy. Thereby, the emulsification processes used in the vaginal drug forms technology can be divided into low-energy (spontaneous emulsification, emulsion phase inversion method, PIM, phase inversion temperature, PIT) and high-energy methods (Figure 3) [62].

Campaña-Seoane et al. obtained water/silicone (W/S) macroemulsions with progesterone or ciprofloxacin via a low-energy formulation preparation process, i.e., mixing the silicone phase with the aqueous phase using Unguator 2 homogenizer [80,81]. In contrast, the formation of W/O/W multiple emulsions using low-energy methods is a two-step process. In the first step inner W/O emulsion is prepared through the addition of aqueous media containing electrolytes into a mixture of oil phase and surfactants (S_mix_) at 80 °C under continuous stirring. In the second step the previously obtained W/O emulsion cooled to room temperature is slowly added to the external aqueous phase that contains hydrophilic surfactant(s) [71,89,101,102,104].

Low-energy spontaneous emulsification generally occurs during vaginal microemulsion or SEDDS formulation [67]. The majority of microemulsion-based vaginal drug forms and some of the nanoemulsions listed in Table 4 can also be obtained using the phase titration method (PTM) or the phase inversion method (PIM). In this approach the aqueous phase is gradually added into the oil and surfactant mixture under continuous stirring. With continuous composition change the surfactant curvature evolves resulting in a W/O to O/W phase change [62,63,64,67,68,78,82,83,84,86,90,94]. PTM is used in the construction of phase diagrams during preformulation research, enabling to establish compositions using which microemulsion can be formed. In this method a mixture of an oil and S_mix_ is slowly titrated with the aqueous phase to obtain a vaginal microemulsion with droplet size in the range of 10–190 nm [78,82,83,84,86].

Mirani et al. [85] obtained tetrahydrocurcumin-loaded vaginal nanoemulsion via the phase inversion temperature (PIT) method. Both phases (oil and water) were initially heated to 45–50 °C followed by mixing and cooling to 25 °C resulting in a nanoemulsion with 130 nm droplets. The PIT method utilizes an alteration in lipophilic properties of nonionic polyoxyethylene-derivative surfactants which can be observed with temperature change—the higher the temperature, the more lipophilic and dehydrated polyoxyethylene surfactant chains become. At the phase inversion temperature (PIT), when the hydrophilic–lipophilic properties of the surfactant are in balance (also called the HLB temperature), a rapid decrease or increase of the temperature enables to obtain a kinetically stable W/O or O/W emulsion, respectively. The PIT method can be easily implemented on the industrial scale, however the stability and polydispersity of the obtained nanoemulsion depends on the experimental conditions and slow heating or cooling of the mixture from HLB temperature enhances droplets coalescence [62,63,64,67,68].

According to Gupta et al. high-energy manufacturing processes are the methods with input energy density higher than 10^8^ W/kg [62]. Among the high-energy emulsification methods used for vaginal nanoemulsion formulation the high-pressure homogenization (pressure range from 50 to 350 MPa), high-speed homogenization and ultrasonication can be distinguished [64,126]. The initial formation of a macroemulsion is required in all high-energy emulsification methods. In the next step macroemulsion droplets are disrupted via high shear forces in high-speed homogenization, turbulence in high-pressure homogenization method or bubbles cavitation in ultrasonication method [62,126]. High-pressure homogenization at 75 MPa preceded by high-speed homogenization allowed dos Santos et al. to obtain a homogenous nanoemulsion with droplets of 281 nm in a short processing time [93]. High pressure homogenization method generates a lot of heat and physical stress which may affect the formulation properties and API stability. The ultrasonication method might be more suitable for thermosensitive drugs, but it is difficult to upscale [62,64,67,68,126]. Low polydispersity of 110–130 nm oil phase droplets has been reported for vaginal nanoemulsions obtained by sonication preceded by low-energy mixing [74,75]. In contrast, nanoemulsion droplets in the range of 58–211 nm (PDI in the range of 0.20–0.34) have been obtained via ultrasonication preceded by high-speed homogenization (10 000 rpm for 25–30 min) [79,92,98]. Examples of nanoemulsions with essential oils manufactured via high-speed homogenization (15,000–17,000 rpm for 20–30 min) enabled to obtain highly homogenous formulations with 68–178 nm oil droplets diameter [95,96]. In nanoemulsion formulation using high-energy methods the surfactants should be mixed with the oil phase prior to the addition of aqueous phase. Mixing emulsion components in a different order results in macroemulsion formation [62,67,68]. Both low- and high-energy methods allow to obtain homogenous nanoemulsions with uniform size distribution of the inner phase droplets which is of importance for formulation of nanoemulsion-based vaginal drug delivery systems [75,78,94,95].

#### 3.2.3. Vaginal Emulsion-Based Drug Delivery Systems Characterization Methods

The 10th Edition of European Pharmacopoeia (Ph. Eur.) includes the monograph ‘Vaginal solution, emulsion and suspensions’ [127] which does not contain detailed requirements concerning their properties including pH, osmolarity, droplet size of the dispersed phase, rheological properties, adhesiveness, spreadability or the release parameters of the active substance. As EVDFs are emerging formulations, in this review we summarized and described research methods that have been proposed for the evaluation of their structural and pharmaceutical properties (see Table 7). In the following sections we propose a unified set of methods which can be used to assess critical parameters of EVDFs. The described characterization methods of vaginal emulsion systems can provide guidance for researchers beginning their studies on these emerging drug delivery systems.

##### pH and Osmolarity

Alkaline preparations administered vaginally may contribute to the development of bacterial infections. Following the above-mentioned WHO recommendations for vaginal lubricants to avoid bacterial superinfections, it is preferable to obtain a formulation with pH below 4.5, i.e., the physiological pH of vagina. This may not be possible for pH sensitive APIs that require higher pH for extended stability or when the benefits of the formulation application exceed the risk of infection as the result of a pH change [45]. In the revised studies only five micro- and nanoemulsion formulations fall within the physiological range of pH values < 4.5 [82,84,86,92,93]. For other reported formulations the pH values were higher and fell within the slightly acidic range, also considered as physiological [78,85,91,94,95,96,97,102]. Some of the formulations pH values exceeded physiological range [89,90,101], creating a risk of bacterial infections. None of the analyzed publications provided information on the osmolarity value of the obtained formulations which, due to the risk of irritation and damage to the vaginal mucosa, should be <1200 mOsm/kg [45]. Gué et al. measured the osmolarity of nanoemulsions intended for parenteral use employing a micro-osmometer and a simple method based on measuring the nanoemulsion’s freezing point depression, which can also be used to determine the osmolarity of EVDF. Osmolarity of nanoemulsions was found to depend on the ratio of lipid and surfactant fractions to water, whereas the concentration of APIs representing BCS (Biopharmaceutical Classification System) class I—paracetamol; class II—ibuprofen, amiodarone hydrochloride, fenofibrate; class IV—ciprofloxacin, had no significant effect on the osmolarity value. In contrast, an increase in the concentration of a substance classified as BCS class III, i.e., ranitidine hydrochloride, caused an increase in the osmolarity of the formulation which should also be taken into account when developing vaginal formulations with low osmolarity <300 mOsm/kg [129].

##### Internal Droplets Measurements, Polydispersity Index and Zeta Potential

Method selection for measuring the dispersed phase droplet size highly depends on the analyzed particles size range. Optical microscopy is most frequently used for emulsions with droplet sizes in the micrometer range, i.e., macroemulsions, as it only allows the observation of particles larger than 200 nm [80,81,89,102,104,130]. The most commonly used method to determine the dispersed phase droplet size of micro- and nanoemulsion-based vaginal dosage forms is the dynamic light scattering (DLS) method, also known as photon correlation spectroscopy, that enables the measurement of droplet sizes in the range from 1 nm to 6 µm [74,75,76,77,78,79,82,83,84,85,86,87,88,90,91,92,93,94,95,96,98,99,103,105]. The accuracy of the obtained results depends on the measurement method and sample preparation, the sample dilution in particular [130]. According to Danaei et al. homogeneous formulations have a low PDI of ≤0.3, moderately homogeneous formulations have PDI in the range of 0.3–0.7 and polydisperse formulations display PDI values > 0.7 [131,132]. The Malvern Zetasizer^®^ is the most commonly used device for both particle size measurements by laser methods and zeta potential measurements by Electrophoretic Light Scattering (ELS). During the zeta potential measurements the device uses the Doppler effect observed in the form of changing scattered laser beam frequency by particles set in motion in the electric field [133]. Although the DLS is frequently used to determine the dispersed phase droplet size, it has several limitations including the necessity of viscous materials dilution as well as the requirement of sample filtration prior to analysis. Furthermore, DLS provides information on hydrodynamic radius of diffusing species rather than the microscopic image of the particles. On the other hand, Transmission Electron Microscopy (TEM) provides high resolution images of nanosize materials, but it requires a particular sample preparation which can change the structure and shape of the dispersed phase droplets [130,133].

##### Viscosity and Adhesion

Viscosity is one of the parameters influencing the degree of the formulation adhesion to the mucosa. In vaginal formulations dynamic viscosity is measured with rheometers and the values are expressed in Pa·s (Pascal-second, SI unit) or P (Puaz, CGS unit); the relation between them is 1 Pa·s = 10 P. Since the measured formulation viscosity depends on the type of equipment including the spindle/cone used and the measurement conditions (i.e., spindle/cone speed and temperature), the experimental viscosity values for the different formulations are only indicative and determine the magnitude of the viscosity range not allowing for a direct comparison of the tested formulations. The viscosity measurement techniques of emulsion-based vaginal formulations and obtained measurements results are collected in Table 7.

**Figure 4:**
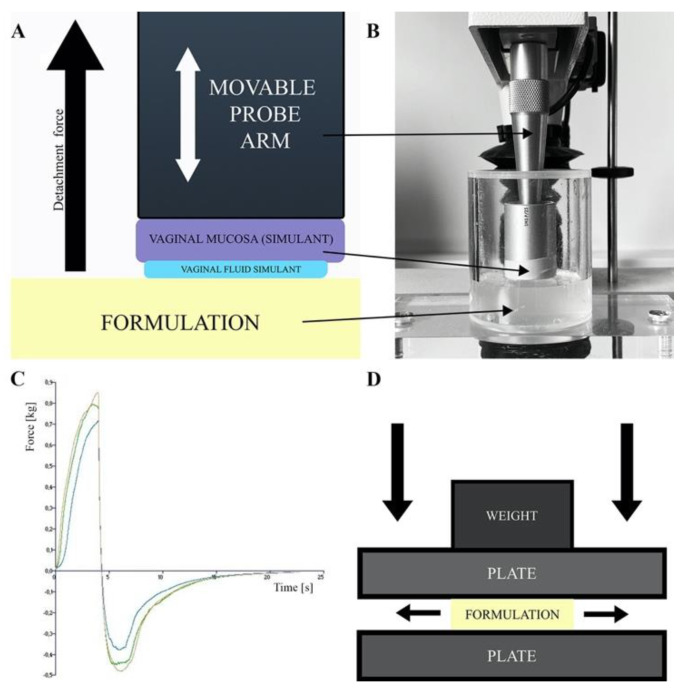
(**A**) Scheme of adhesion measurements by detachment method, (**B**) texture analyzer stable micro system, (**C**) adhesion measurement sample result and (**D**) spreadability test by plate-plate method.

The degree of drug form adhesion to the vaginal mucosa determines its resistance to washing-out by vaginal secretions. In vitro and in vivo methods are used to determine the formulation’s adhesiveness. In vitro methods are based on peel, shear or tensile forces measurements, viscosity difference measurements between the formulation, vaginal secretions and their mixture and flow retention measurements, i.e., the time the formulation remains in contact with the mucous membrane during constant liquid washing [134]. An important factor affecting the comparability of in vitro methods with the real performance of the drug formulation after vaginal administration is the choice of material or tissue used as a model for the vaginal mucosa and the composition of artificial vaginal discharge used in the study [134]. The artificial vaginal discharges are mainly composed of sodium chloride, lactic acid, glucose and additional components such as proteins, electrolytes and buffers dissolved [135]. Their characteristics have been well-described by Tietz and Klein [135]. In vitro adhesion tests measure the tensile strength between the surface of a plate or model tissue attached to the base of a texture analyzer or dynamometer and formulation (see Figure 4A–C) [75,80,81,82,84,86,90,93,94,95,106]. As surface models simulating the human vaginal mucosa the animal tissues (i.e., pig, goat, cow vagina and goat’s skin) are used, as well as synthetic models like cellophane membrane [75,80,81,82,84,86,90,93,94,95,106]. Bachhav and Patravale and Khattab and Ismail [82,84,88] conducted mucoadhesion studies using the Nakamura et al. method [128] which is a modification of the flow retention measurement method. In this method the microemulgels were applied onto an agar plate attached to a USP disintegration test apparatus and subjected to cycles of immersion and emergence in pH = 4.5 buffer until the formulation was washed off entirely of the agar plate.

In the in vivo adhesion studies the measurement of mucoadhesion was based on the visual assessment of the presence of the pigmented nanoemulsion formulation in the rat vagina 24 h after application [94] or the real-time tracking of the localization of the radiolabeled formulation in the animal vagina using PET/CT imaging [80].

Comparison of vaginal formulations’ adhesiveness is challenging due to the differences in measurement devices, measurement conditions and applied vaginal mucosa models. In addition, the results presented as strength expressed as a numerical value are difficult to relate to real vaginal administration conditions. An essential advantage of these studies is the possibility to compare the developed formulations with commercial preparations with known vaginal retention parameters which provides an opportunity to assess the properties of the newly designed formulations.

##### Spreadability

The formulation’s spreadability test determines the increase in the formulation surface area under an external force and enable a comparison of the spreading properties of the investigated formulations as a function of the composition variables and measurement conditions [89,91]. In the case of vaginal formulations, the larger the surface of vaginal mucosa covered by the formulation, the larger the area available for the API penetration. Spreadability, viscosity and adhesion are important in vitro parameters enabling to assess the formulations resistance to the washing-out by vaginal secretions. A plate-plate technique shown in Figure 4D and a texture analyzer are used to assess the spreadability of EVDF [82,84,85,86,89,90,91,95,105]. The changes in surface area covered by investigated formulation as a function of the applied pressure/load force enable a comparison of the spreading properties of the tested formulations as a function of the composition variables and measurement conditions [89,91].

##### In Vitro Drug Release and Permeability Studies

**Figure 5:**
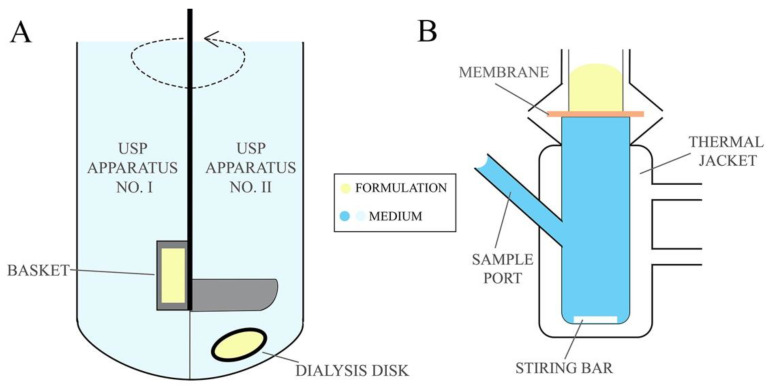
(**A**) USP dissolution apparatus and (**B**) Franz Cells unit.

Drug dissolution studies are used to determine the amount of drug released from the formulation into the acceptor medium over the experimental time. Drug dissolution testing enables to establish drug release kinetic model and the rate at which the API is released from the formulation providing essential information about the pharmaceutical properties of formulations [81]. Permeability studies, on the other hand, determine drug penetration via mucosa enabling to estimate the in vivo performance of the formulation. The permeation studies are frequently designed in comparative manner that allows for the comparison of the newly developed formulation with commercially available products [136]. The release tests are conducted using United States Pharmacopeia (USP) Apparatus No. I and II (Figure 5A) (described in the monograph <711> Dissolution Test [137]), also described in European Pharmacopeia 10.0 as basket apparatus and paddle apparatus, respectively [138], usually for 24 h. Using the USP No. I apparatus the formulations are placed in a basket which is immersed in a vessel with acceptor medium heated to 37 ± 0.5 °C and stirred with predefined rotating rate. In the No. II apparatus, however, the test formulation is placed in a dialysis bag or in a disc covered with a semipermeable membrane immersed below a paddle agitator in a vessel filled with acceptor medium. Citrate and phosphate buffer solutions or simulated vaginal discharge are used as the acceptor medium for pharmaceutical bioavailability studies, while sampling is performed in an automated manner at predefined time points [79,81,82,87,88,90,97]. As an alternative, the drug release/membrane penetration studies from EVDF can be performed using dialysis bags, tubes or chambers immersed in a vessel with mixed and thermostated acceptor medium, e.g., simulated vaginal discharge while samples are withdrawn at predefined time points [74,85,92,98,99,102,103].

The measurements of ex vivo or in vitro permeation of the active substance from the emulsion-based vaginal formulation can be performed with the Franz diffusion cells, illustrated in Figure 5B, through a mucous or synthetic membrane [139]. An important factor affecting the quality of the obtained permeation results is the type of membrane used in the test. In the studies on EVDF, biological tissues simulating conditions of the human vagina and synthetic cellulose membranes (i.e., Visking Medicell Membrane, Filter paper Whatman 41) are employed. As an acceptor medium in permeation tests, citrate and phosphate buffer solutions, a water/ethanol mixture (e.g., for clotrimazole) and simulated vaginal secretions are used. The duration of conducted permeation studies in Franz diffusion cells at 37 °C varied from a few hours to 15 days, with sampling at defined time intervals [80,81,86,89,91,94,106].

In both release kinetics and permeation studies, determination of the released substance concentration in the acceptor medium is usually carried out by HPLC chromatography or UV-VIS spectroscopy.

##### In Vivo Studies

The available in vivo studies evaluate safety and efficacy of emulsion formulations after intravaginal administration in mice, rats, rabbits and pigs. The therapeutic effect after local application of the intravaginal formulation is frequently compared with the effect of systemic administration of the drug, e.g., by oral route [74,75,76,79,80,81,84,87,88,91,92,94,95,96,98,100,102,103]. To date the results from two of the phase I clinical studies (10 and 11 patients respectively) evaluating the safety and therapeutic efficacy of emulsion formulations with clotrimazole and fluconazole in comparison with commercial reference products are available [84,91].

## 4. Emulsion-Based Vaginal Dosage Forms with Drugs from Different Therapeutic Groups—Biological Evaluation and Examples of In Vivo Applications

### 4.1. Antifungal Activity

Vulvovaginal candidiasis is one of the most frequently occurring vaginal fungal infections [140]. The problem affects not only adult women but also children and adolescents with comorbidities such as type 1 diabetes mellitus [141]. A total of 75% of women suffer from vaginal candidiasis at least once in their life. Furthermore, recurrent vulvovaginal candidiasis defined as at least four repeating candida infections per year is an emerging global clinical problem that affects 138 million women every year [142,143]. Among all of the vulvovaginal candidiasis treatment strategies local drug administration has the most advantageous safety and efficiency profile as it reduces the risk of side effects, especially in extreme caution conditions, e.g., pregnancy [144,145].

#### 4.1.1. Antimycotic Azoles

In the treatment of vulvovaginal fungal infections the most abundantly used antifungal group of drugs are azoles being imidazole derivatives [144]. Although azoles have high antimycotic activity, their application is limited due to the insufficient water solubility [146,147]. Emulsion-based antifungal azoles formulations displayed high drug loading capacity, as well as increased drug bioavailability after both oral and topical administration as compared to commercial formulations and neat substances [148,149,150,151,152,153,154,155,156,157].

##### Fluconazole, Clotrimazole

Bachhav and Patravale have developed microemulsion-based gels with fluconazole and with clotrimazole [82,84] which, unlike the reference Candid-V^®^ market gel with clotrimazole, had physiological pH. In vitro release studies of clotrimazole from the final microemulsions showed drug release kinetics similar to Candid-V^®^ gel (Glenmark Pharmaceuticals Limited, Mumbai, India), simultaneously with a greater total amount of the released substance after 10 h of dissolution test. In in vitro antifungal activity tests both microemulsion-based gels with clotrimazole and with fluconazole displayed a larger fungi growth inhibition area as compared to Candid-V^®^ gel (Glenmark Pharmaceuticals Limited, Mumbai, India) [82,84]. After vaginal tolerance study in rabbits, the fluconazole formulation was implemented in a 6-day, double-blind, randomized pilot study with 11 female patients suffering from vaginal candidiasis, divided into a study group (n = 6) treated with fluconazole formulation and control group (n = 5) treated with Candid-V^®^ (Glenmark Pharmaceuticals Limited, Mumbai, India). The study showed similar effects of the fluconazole microemulsion-based gel compared to the Candid-V^®^ gel (Glenmark Pharmaceuticals Limited, Mumbai, India), while reducing by one day the time needed to relieve symptoms in the test group in comparison to the control group [84]. Anticandidal nanoemulsion and W/O/W multiple emulsion with clotrimazole obtained by Soriano-Ruiz et al. had higher antifungal in vitro activity as compared to clotrimazole solution and commercially available 1% and 2% clotrimazole intravaginal creams (Canesten^®^, Bayer, Leverkusen, Germany and Gine-canesten^®^, Bayer, Leverkusen, Germany) [89,91]. The determined minimum inhibitory concentration (MIC) of the evaluated creams and emulsion-based formulations against *Candida glabrata* ATTC 66032 were 7.8125 µg/mL and 0.2441 µg/mL, respectively. Furthermore, the developed formulations displayed enhanced in vitro drug release and ex vivo permeation through the skin as compared to the commercial reference products. In ex vivo studies clotrimazole nanoemulsion displayed four times higher vaginal drug retention and nine times higher theoretical steady-state plasma concentration in comparison to commercial formulations. The results demonstrated high antifungal activity and advantageous pharmacokinetic properties as compared to commercially available clotrimazole creams.

##### Itraconazole

Mirza et al. developed itraconazole nanoemulsion thermosensitive gel using Poloxamer 407 and CP 934 as gelling agents and tea tree oil as the oil phase [94]. Due to the low solubility of the drug in the oils the API was firstly dissolved in chloroform following its evaporative removal from the formulation. The concentration of Poloxamer 407 affected the gelation temperature and mucoadhesive properties of the formulations. Increase in the adhesion and decrease in the gelation temperature was observed with an increasing concentration of Poloxamer 407 in the nanoemulsion-based gel. The optimal pharmaceutical properties of the formulation were obtained at 0.3% CP 934 and 20% of Poloxamer 407 concentration in the emulgel. The developed itraconazole/tea tree oil nanoemulsion-based gel displayed higher antifungal activity as compared to conventional itraconazole or tea tree oil gels, proving the synergetic effect of both ingredients with no toxicity in female rats during in vivo studies. After 14 days of treatment with nanoemulsion-based itraconazole gel female rats were considered cured, while in the vaginal discharge of rats treated with conventional itraconazole gel or tea tree oil gel the concentration of *Candida albicans* cells indicated a still ongoing infection.

##### Sertaconazole

Patel and Patel developed controlled drug release gels based on sertaconazole microemulsion [86]. The drug release kinetics was controlled via changes in polymer concentration. In vitro antifungal activity of optimized formulation against *C. albicans* ATCC 10231 strain was assessed using the cup plate technique and compared to the antifungal activity of commercially available Candid-V^®^ gel (Glenmark Pharmaceuticals Limited, Mumbai, India). After 48 h of incubation the sertaconazole microemulsion-based gel, Candid-V^®^ (Glenmark Pharmaceuticals Limited, Mumbai, India) gel and sertaconazole solution’s inhibition areas were ca. 35, 28 and 29 mm respectively. These results proved a higher antifungal activity of sertaconazole microemulsion-based gel compared to the commercial product and the sertaconazole solution [86].

#### 4.1.2. Nystatin

Song et al. [74] have prepared an oil in water nanoemulsion with nystatin and exopolysaccharide dedicated to the treatment of vulvovaginal candidiasis with sustained-release. The conducted in vitro microbiological test evaluation revealed higher antifungal activity of nystatin/exopolysaccharide nanoemulsion against *C. albicans* cultures and 32 times lower MIC compared to nystatin solutions. In vivo studies also confirmed the synergistic activity of nystatin and exopolysaccharide, increased formulation adhesion to the vaginal mucosa and higher effectiveness in the treatment of vulvovaginal candidiasis than for a nifuratel-nystatin cream. Effective relief of candidiasis symptoms was achieved after 15 days of formulation administration, while the nifuratel-nystatin cream-treated control group still suffered from the infection.

#### 4.1.3. Antifungal Phytoconstituents

Srivastava et al. [95] developed a nanoemulsion-based gel with a natural antifungal agent *Mentha spicata* L. var. virdis aromatic oil (MEO) and Carbopol 940 for the treatment of vaginal candidiasis. The authors concluded that volatility of Mentha essential oil was decreased when incorporated into nanoemulsion droplets providing extended stability of the prepared formulation. The in vivo studies in female proved higher antifungal activity of the obtained MEO nanoemulsion-based gel as compared to a MEO conventional emulgel.

Dos Santos Ramos et al. [75] developed a nanoemulsion with a luteolin-rich *Syngonanthus nitens* (Bong.) extract for vulvovaginal candidiasis treatment. In vivo studies in female Wistar rats have proven a greater antifungal activity of nanoemulsion with phytoconstituents as compared to free phytoconstituents fraction and a commercially available amphotericin B and tetracycline cream. Animals were considered cured after a 6-day treatment with nanoemulsion as compared to a 10-day treatment with cream and free phytoconstituents fraction. In the following study Dos Santos Ramos et al. [93] reported an anticandidal geranium oil nanoemulgel with chitosan as a gelling agent. The chitosan addition increased the mucoadhesive properties of obtained formulations and as a consequence extended their residence time on the vaginal mucosa as compared to a neat nanoemulsion. The optimized nanoemulgel showed a 64-times higher antifungal activity in vitro as compared to a diluted geranium oil.

Gündel et al. [96] presented two nanoemulsions with eucalyptus and lemongrass essential oils as an alternative to azole treatment of vulvovaginal candidiasis. The in vivo studies in mice confirmed antifungal activity of the developed formulations on a par with reference miconazole cream and higher than neat essential oils. The anti-inflammatory effect of the eucalyptus and lemongrass essential oils nanoemulsions was proven in a histopathological analysis after 8 days of treatment. In contrast, mild inflammation in vaginal mucosa tissues was observed in the group treated with a miconazole cream.

Pandit et al. [97] developed lawsone (an antifungal agent obtained from henna) SMEDDS incorporated into easy-to-administer hollow pessaries. Emulsification of lawsone in the formulation provided a higher antifungal activity in vitro as compared to neat lawsone. The hollow pessary can be used for low volumes of concentrated API emulsions providing convenient application and reproducible dosing.

### 4.2. Antibacterial Activity

Apart from fungal infections women, especially in prepubertal age, suffer from bacterial vaginitis and vaginosis. In contrary to candidiasis which is more frequent among sexually active women, bacterial infections in children and adolescent virgins are related to improper hygiene. Atrophic vaginal mucosa is prone to pathogens due to lower levels of estrogen, not fully grown bacterial flora resulting in alkaline pH and deficiency of defensive factors such as bacteriocins and hydrogen peroxide [37,158,159,160,161,162,163,164,165].

#### 4.2.1. Antibiotics and Chemotherapeutics

A group of Otero-Espinar [80] developed controlled release vaginal W/S emulsions with a model BCS IV class drug, i.e., ciprofloxacin. The radiolabeled W/S formulation after administration in female rats was resistant to wash-out by vaginal discharges for 90 min as monitored using PET/CT imaging. The real-time observation revealed systemic absorption of ciprofloxacin as the drug was distributed to the urinary bladder. After topical administration a higher ciprofloxacin concentration in uterine tissues as compared to blood concentration was reported. Furthermore, six hours after the intravaginal administration of the W/S emulsion ciprofloxacin concentration in uterine tissues was comparable to the drug concentration achieved via an intraperitoneal injection. High drug concentrations in the genital tissues were observed for up to 24 h after vaginal administration of the W/S emulsion. The presented approach may be used in future treatment of urinary tract infections in patients unable to administer the drug orally or experiencing the side effects after oral administration.

Atinderpal et al. [79] developed a ciprofloxacin and green tea Polyphenon 60 nanoemulsion with confirmed activity against ESBL and MBL bacteria. The vaginally administered nanoemulsion showed higher biodistribution, drug bioavailability and blood concentrations compared to the oral route in in vivo studies. After vaginal administration high renal and urinary bladder concentrations of ciprofloxacin have been observed which should be considered in future administration of proposed formulation in patients with renal impairment [79]. Studies of both Otero-Espinar et al. and Atinderpal et al. have proven that ciprofloxacin applied as a vaginal emulsion undergoes direct vagina-to-uterus transport known as the first uterine pass effect [20,79,80].

Özer et al. [102] developed multiple W/O/W emulsions with metronidazole and ornidazole for the potential treatment of vaginal bacterial infections. In vitro studies demonstrated that the release kinetics of oil-encapsulated chemotherapeutics were similar compared to the drug release from the continuous phase. However, alkaline dissolution media increased the release of metronidazole and ornidazole from both the internal and external emulsion phases. This can prove advantageous in the treatment of bacterial vaginitis where higher pH of vaginal secretions is observed, enabling for increased chemotherapeutic release rate and, as a consequence, faster relief of the infection symptoms [166,167]. After application of a multiple emulsion with radioisotope-labeled compounds in rabbits, a faster absorption of metronidazole than ornidazole from the vaginal epithelium was observed. Both APIs encapsulated in a W/O/W multiple emulsion have proven effective against local bacterial infections with only a minor increase in the blood concentration and, at the same time, a small substance increase in blood concentrations in rabbits was observed, confirming the local effect of the drug.

#### 4.2.2. Antiseptics

Tedajo et al. [101] developed a vaginal W/O/W emulsion as a formulation for potential treatment of female reproductive tract infections, mainly caused by *Escherichia coli, Staphylococcus aureus* and *C. albicans*. Obtained multiple emulsions allowed the simultaneous administration of benzalkonium chloride, octadecylamine and lactic acid without the risk of pharmaceutical incompatibility, demonstrating at least one-year storage stability [101,168,169]. Although the optimized multiple emulsion showed an increased microbicidal efficacy against *S. aureus* and *C. albicans* compared to the free 0.2% benzalkonium chloride solution, the formulation displayed lower microbicidal activity against *E. coli* compared to an antiseptic reference solution.

Tedajo et al. [104] investigated the release of benzalkonium chloride and chlorhexidine digluconate encapsulated in external and internal aqueous phases of a W/O/W multiple emulsion, respectively. The synergistic effect of the combination of chlorhexidine digluconate and benzalkonium chloride in the form of a multiple emulsion against *S. aureus* and *E. coli* was confirmed in vitro. Prior to vaginal administration of W/O/W emulsions the water-dilution is recommended, which provides hypotonic conditions facilitating APIs release from the internal phase of the emulsion as a result of the swelling-breakdown process. Additionally the encapsulation of labile substances, such as light-sensitive chlorhexidine digluconate, to the internal phase of multiple emulsions provided extended API stability in emulsion-based formulations [104].

#### 4.2.3. Emulsions with Phytoconstituents

Abu-Azzam and Nasr [83] have developed an intravaginal microemulsion with an anti-inflammatory phloretin phytoconstituent for potential treatment of vaginitis. The formulation showed an enhanced anti-inflammatory activity compared to the free phloretin in cell cultures, achieving similar efficacy to diclofenac sodium. The obtained initial results indicated that a vaginal microemulsion with phloretin may be a potential therapeutic alternative to non-steroidal anti-inflammatory drugs, while its safety and efficacy needs to be confirmed in vivo in the future.

For the potential treatment of *E. coli* vaginal infections Kaur et al. obtained two nanoemulsion-based gels composed of green tea Polyphenon 60 mixed with curcumin or cranberry [92,98]. Vaginally administered formulation of radiolabeled Polyphenon 60 in female rats showed higher distribution in the kidney and urinary bladder as compared to vaginal application of drug solutions or orally administered nanoemulgels. The studies of Kaur et al., confirmed that intravaginal administration of Polyphenon 60 with curcumin or cranberry nanoemulsion might be a promising intravaginal and urinary tract infections treatment alternative to the conventional oral administration of antibiotics [92,98].

### 4.3. Contraceptive and Sexually Transmitted Diseases Prevention

At the end of the 20th and the beginning of the 21st centuries there was a strong interest in the microemulsion-based gels used as vaginal spermicides. D’Cruz and Uckun, in the review article, referred to the safety and spermicidal activity of emulsion-based gels GM-4 and GM-144 compared to commercially available nonoxynol-9 (N-9) gel [77]. After the analysis of in vivo toxicity and spermicidal activity tests in rabbits and mice, D’Cruz and Uckun concluded that microemulsion-based gels are less toxic and more effective than the commercially available intravaginal contraceptive [77]. D’Cruz and Uckun [76] developed intravaginal microemulsion-based gel with contraceptive chelated vanadocene as an alternative to popular N-9 gel that was reported to have a toxic effect on the vaginal epithelium and questionable spermicidal efficacy [113,170,171]. The proposed microemulsion gel formulation revealed promising contraceptive activity in vivo in rabbits and in pigs [76].

Mirani et al. proposed microemulsion-based gel with tetrahydrocurcumin (THC) as a vaginal microbicide in the prophylaxis of HIV infections [85]. The final formulation exhibited pseudoplastic behavior and comparable viscosity to Durex^®^ gel, as well as stable and resistant to sexual intercourse stress THC release profile [85].

D’Cruz et al. [172] described the usage of the self-emulsifying gel—Conceival for intravaginal delivery of lipophilic anti-HIV drugs. Further, McConville and Friend [99] developed SMEDDS-filled capsules for the vaginal administration of another HIV microbicide—a thiocarboxanilide non-nucleoside reverse transcriptase inhibitor (UC781). Prepared by McConville and Friend SMEDDS displayed rapid dispersion and enhanced UC781 release in sink conditions as compared to poorly soluble UC781 powder. The authors concluded that further studies are required for the optimization of the formulation size and UC781 content to tailor properties to intravaginal application.

Köllner et al. [105] developed SEDDS for vaginal delivery of curcumin as a potential anti-HPV agent. Curcumin is practically insoluble in water and has a high binding affinity to mucus, which hinders its absorption after administration on the mucosa. In vitro permeation tests of SEDDS with curcumin showed that ≈15% of the curcumin load penetrates mucus within 3 and ≈35% within 24 h after application proving SEDDS as potential intravaginal carriers for lipophilic, poorly soluble drugs.

### 4.4. Other Diseases and Conditions

#### 4.4.1. Pre-Term Birth Prevention and Hormonal Therapy

The group of Otero-Espinar [81] proposed W/S emulsions with progesterone as the potential formulations for intravaginal drug administration. Rheological tests revealed higher bioadhesion and higher resistance of emulsion formulation to simulated vaginal discharges in relation to commercially available intravaginal gels. In vivo studies in postmenopausal and young rats have shown that after the application progesterone concentration in the uterine tissue was significantly higher in the group treated with the intravaginal W/S emulsion as compared to the group treated with commercial Crinone^®^ (Merck KGaA, Darmstadt, Germany). During in vivo studies the progesterone accumulation in the uterus tissue was also observed due to the first uterine pass effect.

Patki et al. [87] obtained SNEDDS with 17-α hydroxyprogesterone caproate in the form of the solid vaginal tablet, providing a single-dose formulation that is easy to use and less invasive as compared to the injections. The dissolution rate of 17-α hydroxyprogesterone from the vaginal tablets was significantly higher as compared to free drug. The investigated tablets released 97% of hormone within 120 min of the dissolution test. In vivo studies in Swiss Webster mice showed that intravaginal administration of 17-α hydroxyprogesterone vaginal tablets with SNEDDS reduced the rate of pre-term births, presenting a safe alternative for hormonal drug injections.

Giusto et al. [88] developed SNEDDS-based vehicle for a Sphingosine kinase (SphK) inhibitor used in the prevention of lipopolysaccharide-induced pre-term birth in mice. The obtained SNEDDS formed a gel in contact with simulated vaginal discharge in situ without signs of precipitation. In the dissolution test, 94% of the drug was released from SNEDDS formulation within 30 min of the experiment as compared to less than 1% of neat API dissolved in the same time. In vivo studies in mice demonstrated significantly smaller number of pre-term births and steady uterus SphK concentration after SNEDDS application.

#### 4.4.2. Tumors and Autoimmunological Diseases

Frank et al. [78] obtained nanoemulsion with anti-HPV drug imiquimod for the treatment of cervical cancer. In vitro analysis of substance permeability confirmed that imiquimod in nanoemulsion formulation was able to penetrate porcine vaginal mucosa with a lower rate than free imiquimod, which in planned indication might be beneficial due to reduced vaginal irritation and risk of adverse effects. Additionally, imiquimod encapsulated in the emulsion-based formulation reduced the growth of cervical carcinoma cell line SiHa in vitro, proving its potential in the treatment of cervical cancer.

Wang et al. [103] developed a new intravaginal W/O/W multiple SMEDDS incorporated in the thermosensitive gel for the delivery of small nucleic acid in a gene-silencing anti-HPV therapy. Authors have decided to encapsulate siRNA into the inner aqueous phase of W/O/W multiple SMEDDS to improve the stability of a labile active ingredient. Multiple W/O/W SMEDDS loaded with siRNA in contrast to other transfection carriers prevented inflammatory reactions in both in vitro and in vivo studies.

## 5. Conclusions and Future Perspective

The development of safe and effective emulsion-based multicompartment vaginal drug formulations should acknowledge the anatomy and physiology of a vagina. The rheological properties, mucoadhesion, pH value, the droplet size of a dispersed phase and an effective dose should be carefully optimized to achieve prolonged retention time in the vaginal environment. The reported research is primarily focused on the identification of optimal formulation properties related to the application site characteristics. Within the emulsion-based multicompartment vaginal drug carriers one can distinguish macro-, micro-, nano- and multiple emulsions as well as SEDDS and emulgels. Most commonly used lipophilic excipients include fatty acid ester derivatives and among the surfactants polysorbates and castor oil polyethylene glycol derivatives are used. Combination of these excipients is selected according to API solubility studies of emulsion-based formulations with higher drug capacity, small size of dispersed droplets and low or moderate polydispersity.

The analysis of vaginal formulation testing methods indicates variability in the researchers’ approach to the selection of methodologies for the evaluation of formulation properties, e.g., type of applied method and the preference of apparatus and materials used. This results in difficulties in comparison between the properties and parameters of similar formulations reported by different groups. It would be advantageous to define a range of optimal parameter values for a specific type of formulation and a methodology for their assessment. The comprehensive evaluation of the in vitro physicochemical and pharmaceutical properties is important to explain clinical observations, enabling the selection of the optimal formulation as a consequence.

Based on the revised reports it can be concluded that Emulsion-based Vaginal Dosage Forms are promising drug carriers for local and systemic application of antimicrobial and anticancer agents. Moreover, their use in the prevention of sexually transmitted diseases and premature births achieved high therapeutic efficacy and a favorable safety profile. EVDF, due to the easily adjustable properties, i.e., method of administration, low volume, adhesiveness, viscosity, selective local drug activity, can also be considered as vaginal pediatric formulations. Furthermore, the presented review has demonstrated that there is a need for randomized clinical trials that would confirm the therapeutic benefits of the most promising EVDF formulations.

## Figures and Tables

**Table 1:** API and formulation properties imperative to vaginal drug delivery forms development [14,44,45,46,47,48,49,50,51,52,53,54,55,56,57].

	Properties
API	low molecular weight hydrophilic or lipophilic compounds
Formulation	water-soluble bioadhesive reduced hydrophobic interactions (details in Section 3) increased viscosity or thermogelling properties easy administration pH ≈ 4 osmolarity < 1200 mOsm/kg, ideally < 380 mOsm/kg inner phase particle/droplets size in the range: ○ 100–150 nm—formulations intended to act within the vaginal mucus ○ 200–500 nm—formulations intended to penetrate through the mucus to the vaginal epithelium ○ >1000 nm—formulations intended to act on the surface of vaginal mucus

**Table 2:** Classification and characteristic of the emulsion-based dosage form [58,59,60,61,62,63,64,65,66,67,68,69,70,71,72].

Type of Formulation	Macroemulsion	Microemulsion	Nanoemulsion	Multiple Emulsion	SEDDS ^a^
**Appearance**	Milky	Transparent	Translucent or transparent	Milky	Depending on the vehicle ^b^
**Droplet size**	>500 nm	Typically < 1000 nm	<500 nm	>1000 nm	<100 nm
**Droplet shape**	Spherical	Spherical and non-spherical	Spherical	Multiple droplets	Spherical and non-spherical ^a^
**Polydispersity**	Often high	Often low (single, narrow distribution peak)	Low or moderate (single or multiple distribution peaks)	Often high	Often low ^a^
**Stability**	Thermodynamically and kinetically unstable	Thermodynamically stable	Kinetically stable	Often thermodynamically and kinetically unstable	-
**Manufacturing methods**	High and low-energy methods	Spontaneous formation	High and low-energy methods	Two-steps low-energy process	Spontaneous formation ^a^

^a^ This column covers properties of SMEDDS and SNEDDS listed in Section 3.1, data are given for formulations diluted with aqueous media; ^b^ formulation properties before a dilution with aqueous media.

**Table 3:** Excipients used as the components of emulsion-based vaginal drug formulations [74,75,76,77,78,79,80,81,82,83,84,85,86,87,88,89,90,91,92,93,94,95,96,97,98,99,100,101,102,103,104,105,106].

Excipients	Group/Function	Applied Excipient
Oil phase	Mineral oils	Paraffin oil/White Vaseline
	Vegetable oils and essential oils	Copaiba oil, Eucalyptus essential oil, Geranium oil, Lemongrass essential oil Mentha essential oil, Soybean oil, Tea Tree oil
	Sterols	Cholesterol
	Phospholipids	Phospholipon 90G (soybean lecithin at 90% of phosphatidylcholine)
	Fatty acids	Oleic acid
	Fatty acid monoesters	Capryol 90 (propylene glycol monocaprylate), Cetyl palmitate, Glycerol monolaurate, Isopropyl myristate, Monoglycerides of caprylic acid
	Fatty acid diesters/triesters	Captex 300 (medium-chain triglyceride of caprylic and capric acid), Labrafac lipophile (medium-chain triglycerides of caprylic and capric acid), Labrasol (PEG-8 caprylic/capric glycerides), other undefined medium chain triglycerides
	Alkene derivates	Parleam (Hydrogenated polyisobutene)
	Organosilicon compounds	Cyclomethicon tetramer, Cyclomethicon pentamer
Surfactants	Non-ionic surfactants	Polysorbates: Tween 20 (polysorbate 20), Tween 80 (polysorbate 80) Sorbitan esters: Span 60, Span 80 PEG derivatives: Gelucire 44/13 (mono/dri/triglycerides and PEG-32 mono- and diesters of lauric acid), Labrasol (PEG-8 caprylic/capric glycerides), Kolliphor EL/Cremophor EL (PEG-35 castor oil), Kolliphor HS (Macrogol (15)-hydroxystearate), Kolliphor RH 40/Cremophor RH 40 (PEG-40 castor oil) Polyoxyethylene derivatives: Brij 20 (polyoxyethylene (20) cetyl ether) Polyoxypropylene derivatives: Pluronic F68 (Poloxamer 188), Pluronic F127 (Poloxamer 407)
	Amphoteric surfactants	Amino acid derivatives: Tego Betain F (Cocamidopropyl Betaine)
	Cationic surfactants	Amins: Cetylpyridinium chloride
	Other surfactants	Organosilicon compounds: Abil WE 09 (polyglyceryl-4 isostearate; Cetyl PEG/PPG-10/1 dimethicone; hexyl laurate), Abil EM 90 (Cetyl PEG/PPG-10/1 Dimethicone) Bacterial saccharides: Exopolysaccharide from *B. vallismortis* WF4 strain (mannose/glucose/xylose/arabinose)
Cosurfactants	Alcohols	Ethanol, glycerol, propylene glycol, transcutol P (2-(2-ethoxyethoxy)ethanol)
	Phospholipids	Soy phosphatidylcholine
	Fatty acids and their monoesters	Caprylic acid, Capryol 90 (propylene glycol monocaprylate)
	PEGs and PEGs derivatives	Labrasol (PEG-8 caprylic/capric glycerides), PEG 200, PEG 300, PEG 400
	Polysorbates	Tween 20, Tween 80
Other	Gelling agents	Carbomers: CP 934 (Carbopol 934), CP 940 (Carbopol 940), CP ETD 2020 (Carbopol ETD 2020), CP U 10 NF (Carbopol Ultrez 10 NF), Tego Carbomer 341 Polyoxypropylene derivatives: Pluronic F127 (Poloxamer 407) Polysaccharides: Chitosan, HPMC (hydroxypropyl methylcellulose), NaCMC (sodium carboxymethyl cellulose), Xantural (XG, xanthan gum)
	Preservatives	Benzyl alcohol, Chlorocresol, Methylparaben, Sodium benzoate
	pH regulators	Lactic acid, Phosphate buffer, Triethanolamine
	Electrolytes	Magnesium sulphate, Sodium chloride
	Humectants	Propylene glycol, PEG 200

**Table 4:** Vaginal macroemulsions, microemulsion- and nano-emulsion-based dosage forms—composition, characteristic properties and manufacturing methods.

API (Indication)	Formulation	Oil Phase/Surfactant/Cosurfactant/Others	Particle Size (nm)	PDI	Zeta Potential (mV)	Manufacturing Method	Ref.
**Vaginal macroemulsions**							
Benzydamine (Antibacterial/Anti-inflammatory)	Emulgel	white Vaseline, paraffin/n.a./n.a./Water phase: NaCMC, glycerol, citrate buffer	n.a.	n.a.	n.a.	Mixing	[106]
Progesterone (n.a.)	W/S emulsion	cyclomethicone pentamer/Abil WE 09/glycerol/Sodium chloride	1000–3000	n.a.	n.a.	Mixing	[81]
Ciprofloxacin (Antibacterial)	W/S emulsion	cyclomethicone pentamer ortetramer/Abil WE 09/glycerol/ Sodium chloride	2230–2540	n.a.	n.a.	Mixing	[80]
**Vaginal microemulsion**							
- (Contraceptive)	Microemulgel	Captex 300/Cremophor EL, Phospholipon 90 G, Propylene Glycol/PEG 200/Seaspan carrageenan, Viscarin carrageenan, Sodium benzoate	30–80	n.a.	n.a.	n.a.	[100]
- (Contraceptive)	Microemulgel	Captex 300/Cremophor EL, Pluronic F68, Phospholipon 90G, Propylene glycol/Xanthan gum, Sodium benzoate	30–80	n.a.	n.a.	n.a.	[77]
Vanadocene (Contraceptive)	Microemulgel	Captex 300, Phospholipon 90G/Cremophor EL, Pluronic F68/Xanthan gum	30–80	n.a.	n.a.	n.a.	[76]
Fluconazole (Antifungal)	Microemulgel	Capryol 90/Cremophor EL/Benzyl alcohol, chlorocresol, CP ETD 2020	24	0.98	n.a	Mixing	[84]
Clotrimazole (Antifungal)	Microemulgel	Capryol 90/Cremophor EL/Benzyl alcohol, chlorocresol, CP ETD 2020	48	0.75	n.a	Mixing	[82]
Sertaconazole (Antifungal)	Microemulgel	Oleic Acid/Tween 80/Propylene glycol/CP 940	26	0.55	0.26	Mixing, dissolving API under ultrasonication	[86]
Tetrahydro-curcumin (Vaginal microbicide, HIV protection)	Microemulgel	Gycerol monolaurate/Tween 20/Transcutol P/CP U 10 NF, triethanolamine	130	0.18	n.a.	Low-energy (mixing and heating)	[85]
Phloretin (Anti-inflammatory)	Microemulsion	Oleic acid/Tween 20/Ethanol	11	n.a	n.a	Mixing	[83]
**Vaginal nanoemulsion**							
Itraconazole (Antifungal)	Nanoemulgel	Tea tree oil/Tween 20/Labrasol/CP 934, Poloxamer 407	42	0.12	−44	Low-energy method (mixing)	[94]
Oxiconazole (Antifungal)	Nanoemulgel	Isopropyl myristate/Cremophor EL/Ethanol/HPMC or XG or CP 934	26	0.55	−34	Low-energy method (mixing)	[90]
Clotrimazole (Antifungal)	Nanoemulsion	Labrafac lipophile/Labrasol/Capryol 90/Propylene glycol (aqueous phase)	153–186	0.37–0.85	−15–−1	Low-energy method (mixing, heating), High-energy method (sonication)	[91]
Polyphenon 60, Curcumin (Antibacterial)	Nanoemulgel	Soybean oil/Tween 20/Propylene glycol/Chitosan	211	0.34	−33	Low-energy method (mixing), High-energy method (high-speed homogenization and ultrasonication)	[98]
Polyphenon 60, cranberry (Antibacterial)	Nanoemulgel	Oleic acid/Tween 20/Glycerol/Chitosan, lactic acid	58	0.20	−16	Low-energy method (mixing), High-energy method (high-speed homogenization and ultrasonication)	[92]
Mentha essential oil (Antifungal)	Nanoemulgel	Mentha essential oil/Tween 80/PEG 400/CP 940, methylparaben, triethanolamine	178	0.18	−32	High-energy method (high-speed homogenization)	[95]
Nystatine (Antifungal)	Nanoemulsion	Paraffin oil/Exopolysaccharide/PEG 400	131	0.08	−40	Low-energy method (mixing), High-energy method (ultrasonication)	[74]
Ciprofloxacin, Polyphenon 60 (Antibacterial)	Nanoemulsion	Labrasol/Cetylperidinum chloride/Glycerol	151	0.20	55	Low-energy method (mixing), High-energy method (high-speed homogenization and ultrasonication)	[79]
Geranium oil (Antifungal)	Nanoemulgel	Geranium oil/Span 80/Tween 20/Chitosan	281	0.32	53	High-energy method (high-speed and high-pressure homogenization)	[93]
*Syngonanthus nitens* (Bong.) extract (Antifungal)	Nanoemulsion	Cholesterol/Brij 20/Soy phosphatidylcholine/Chitosan, phosphate buffer	111	0.30	2	Low-energy method (mixing), High-energy method (sonication)	[75]
Imiquimod (Cancer treatment)	Nanoemulsion	Copaiba oil/Span 60/Tween 80	190	0.11	n.a.	Low-energy method (mixing and solvent evaporation)	[78]
Eucalyptus essential oil (Antifungal)	Nanoemulsion	Eucalyptus essential oil/Polysorbate 80/Sorbitan monooleate	68	0.18	−9	High-energy (high-speed homogenization)	[96]
Lemongrass essential oil (Antifungal)	Nanoemulsion	Lemongrass essential oil/Polysorbate 80/Sorbitan monooleate	90	0.21	−8	High-energy (high-speed homogenization)	[96]

n.a.—information not available.

**Table 5:** Vaginal multiple emulsions—composition, characteristic properties and manufacturing method.

API(s) (Indication)	Formulation	Oil Phase/Lipophilic Surfactant/Hydrophilic Surfactant/Other	Particle Size (nm)	PDI	Zeta Potential (mV)	Manufacturing Method	Ref.
W_1_: benzalkonium chloride O: octadecylamine W_2_: lactic acid (Antibacterial)	Multiple emulsion	Parleam/Abil EM 90/Poloxamer 407	>5000	n.a.	n.a.	Two-step process	[101]
W_1_: benzalkonium chloride W_2_: chlorhexidine (Antibacterial)	Multiple emulsion	Parleam/Abil EM 90/Poloxamer 407/Sodium chloride	>5000	n.a.	n.a	Raynal method [71]	[104]
W_1_: metronidazole W_2_: Ornidazole (Antibacterial)	Multiple emulsion	Parleam/Abil EM 90/Poloxamer 407/Magnesium sulphate	>8000	n.a.	n.a.	Raynal method [71]	[102]
O: Clotrimazole (Antifungal)	Multiple emulsion-based gel	Labrafac lipophile, Cetyl palmitate/Abil EM 90, Span 60/Cocamidopropyl Betaine/Tego Carbomer 341, Sodium chloride	>29,000	n.a.	−55	Modification of Raynal method [71]	[89]

n.a.—information not available, O—oil phase, W1—internal aqueous phase, W2—external aqueous phase.

**Table 6:** Vaginal self-emulsifying drug delivery systems—composition, characteristic properties and manufacturing method.

API (Indication)	Formulation	Oil Phase/Surfactant/Cosurfactant/Others	Particle Size (nm)	PDI	Zeta Potential (mV)	Manufacturing Method	Ref.
UC 781 (HIV-protection)	SMEDDS	Mono- and diglycerides of caprylic acid/Cremophor RH40/PEG 300	13	0.25	32	Mixing	[99]
Curcumin (HPV-protection)	SNEDDS	Medium chain triglycerides/Cremophor RH40/PEG 200, Caprylic acid, Tween 80	38	0.35	−1	Mixing	[105]
17-α hydroxyprogesterone (Pre-term births prevention)	Solid-state SNEDDS Vaginal tablet	Captex 300/Kolliphor HS/Polyvinyl alcohol, calcium silicate, microcrystalline cellulose, Kollidon CL, Magnesium stearate	50	0.09	−7	Mixing, tablet formation	[87]
The SphK inhibitor (Pre-term births prevention)	SNEDDS	Captex 300/Kolliphor HS/Dimethyl-acetamide	37	0.05	−5	Mixing	[88]
W_1_: siRNA (Gene silencing)	Multiple microemulsion SEDDS gel	Medium chain triglycerides/Lipophilic: Cremophor RH40, Span 80; Hydrophilic: Cremophor RH 40/Lecithin (hydrophilic and lipophilic)/‘thermosensitive gel’ ^a^	167	0.18	−7	Two-step process	[103]
Lawsone (Antifungal)	SMEDDS hollow pessary	Capryol 90/Gelucire 44/14/Tween 80/Ovucire WL3460, beeswax	12	0.27	−11	Mixing	[97]

^a^—based on information provided by the authors, gel composition was not specified, n.a.—not available, W_1_—internal aqueous phase.

**Table 7:** Comparison of parameters and research methods using in evaluation of emulsion-based vaginal dosage forms properties.

API(s) (Formulation)	pH	Droplet Size	Viscosity ^c^ (Pa·s)	Spreadability	Bioadhesion	In Vitro Release/Permeability	In Vivo Studies	Ref.
**Vaginal macroemulsions**								
Benzydamine (Emulgel)	-	-	Plate-plate (100–700)	-	in vitro, T/DF, porcine VM	Franz cells	-	[106]
Progesterone (W/S * emulsion)	-	MS	Cone-plate (21.2–186.6)	-	in vitro, T/DF, bovine VM, GTL	USP II/Franz cells	rats	[81]
Ciprofloxacin (W/S * emulsion)	-	MS	Cone-plate (1.4–17.0) (1.5–14.0)	-	in vitro, T/DF, GTL	Franz cells and in vivo	rats	[80]
**Vaginal microemulsion**								
- (Microemulgel)	-	DLS	+ ^a^	-	-	-	rabbits	[100]
- (Microemulgel)	-	DLS	+ ^a^	-	-	-	rabbits	[77]
Vanadocene (Microemulgel)	-	DLS	Results n.a.	-	-	-	rabbits, pigs	[76]
Fluconazole (Microemulgel)	4.5 ^b^	DLS	Spindle (9800 at 5 rpm)	P-P	in vitro, NM, agar plate	-	rabbits, 11 female patients	[84]
Clotrimazole (Microemulgel)	4.5 ^b^	DLS	Spindle (9000 at 5 rpm)	P-P	in vitro, NM, agar plate	Modified Apparatus No. 1 USP 23	-	[82]
Sertaconazole (Microemulgel)	4.2 ^b^	DLS	+ (2.0)	P-P	in vitro, T/DF, goat VM	Franz cells	-	[86]
Tetrahydrocurcumin (Microemulgel)	6.0 ^b^	DLS	Spindle (11.5 at 5 rpm)	TA	-	Dialysis bag	-	[85]
Phloretin (Microemulsion)	-	DLS	-	-	-	-	-	[83]
**Vaginal nanoemulsion**								
Itraconazole (Nanoemulgel)	5.5 ^b^ (nanoemulsion)	DLS	Spindle (0.91)	-	in vitro, T/DF, CM, in vivo (rats)	Franz Cells	rats	[94]
Oxiconazole (Nanoemulgel)	6.9 ^b^ (gel with HPMC)	DLS	Cone-plate (8.43 at 50 rpm for gel with HPMC)	P-P	in vitro, NM, animal vagina	USP II	-	[90]
Clotrimazole (Nanoemulsion)	5.7 ^b^	DLS	Cone-plate (0.041–0.042 at 100/s)	P-P	-	Franz Cells	10 women—skin tolerance	[91]
Polyphenon 60, Curcumin (Nanoemulgel)	-	DLS	+ (0.66–141)	-	-	Dialysis bag	rats	[98]
Polyphenon 60, cranberry (Nanoemulgel)	3.7 ^b^	DLS	+ (>141 at 0.01/s)	-	-	Dialysis cells	rats	[92]
Mentha essential oil (Nanoemulgel)	5.2 ^b^	DLS	Spindle (24.8)	TA	in vitro	-	mice	[95]
Nystatine (Nanoemulsion)	-	DLS	Spindle (0.12)	-	-	Dialysis bag	mice	[74]
Ciprofloxacin, Polyphenon 60 (Nanoemulsion)	-	DLS	-	-	-	USP II	rats	[79]
Geranium oil (Nanoemulgel)	4.4 ^b^	DLS	Spindle (0.4–0.5 at 50/s–0.01/s)	-	in vitro, T/DF, porcine VM	-	-	[93]
*Syngonanthus nitens* (Bong.) extract (Nanoemulsion)	-	DLS	Cone-plate	-	in vitro, T/DF, porcine VM	-	rats	[75]
Imiquimod (Nanoemulsion)	6.0 ^b^	DLS	-	-	-	Franz cells	-	[78]
Eucalyptus essential oil (Nanoemulsion)	5.3 ^b^	DLS	-	-	-	-	mice	[96]
Lemongrass essential oil (Nanoemulsion)	4.6 ^b^	DLS	-	-	-	-	mice	[96]
**Vaginal multiple emulsions**								
W_1_: benzalkonium chloride O: octadecylamine W_2_: lactic acid (Multiple emulsion)	7.8 ^b^	MS, GA	Cone-plate (3.2 at 100/s)	-	-	-	-	[101]
W_1_: benzalkonium chloride W_2_: chlorhexidine (Multiple emulsion)	-	MS	Cone-plate (Isosmotic condition: 0.003 at 100/s)	-	-	Conductometric (NaCl as a marker)	-	[104]
W_1_: metronidazole W_2_: ornidazole (Multiple emulsion)	W_1_: 5.7 ^b^ W_2_: 6.0 ^b^	MS	-	-	-	Dialysis tube	rabbits	[102]
O: Clotrimazole (Multiple emulsion-based gel)	6.5 ^b^	LD	parallel plate-plate (0.29 at 100/s)	P-P	-	Franz cells	-	[89]
**Vaginal Self-Emulsifying Drug Delivery Systems**								
UC 781 (SMEDDS)	-	DLS	-	-	-	Dialysis bag (balloon)	-	[99]
Curcumin (SNEDDS)	-	DLS	Plate-plate (116.3)	OM	-	Transwell chambers	-	[105]
17-α hydroxyprogesterone (Solid-state SNEDDS Vaginal tablet)	-	DLS	-	-	-	USP II	mice	[87]
The SphK inhibitor (SNEDDS)	-	DLS	Spindle (0.2-fold dilution: 0.53 0.4-fold dilution: 4.8 at 20 rpm)	-	-	USP II	mice	[88]
W_1_: siRNA (Multiple emulsion SEDDS gel)	-	DLS	-	-	-	Dialysis bag	mice	[103]
Lawsone (SMEDDS hollow pessary)	4.2–4.8 ^b^	DLS	Spindle (0.956–1.023)	-	-	USP I	-	[97]

(-)—test not reported to perform; (+)—test reported to perform, method details not specified; *—water in silicon emulsion; ^a^—authors reported that viscosity of emulgels were in the range of 1–10 Pa·s; ^b^—potentiometric method; ^c^—method used for viscosity measurement and provided value; CM—cellophane membrane; DLS—dynamic light scattering; GA—granulometric analysis; GTL—goat tanned leather; LD—laser diffractometry; MS—microscopy; NM—Nakamura method [128]; OM—optical method; P-P—plate-plate method; results n.a.—test performed, results not available in a paper; TA—texture analyzer; T/DF—tensile/detachment force; USP I—USP dissolution apparatus No. I; USP II—USP dissolution apparatus No. II.; VM—vaginal mucosa.

## Data Availability

Not applicable.
